# Using multi-locus allelic sequence data to estimate genetic divergence among four *Lilium* (*Liliaceae*) cultivars

**DOI:** 10.3389/fpls.2014.00567

**Published:** 2014-10-20

**Authors:** Arwa Shahin, Marinus J. M. Smulders, Jaap M. van Tuyl, Paul Arens, Freek T. Bakker

**Affiliations:** ^1^Wageningen UR Plant Breeding, Wageningen University and Research CentreWageningen, Netherlands; ^2^Biosystematics Group, Wageningen UniversityWageningen, Netherlands

**Keywords:** *Lilium*, allelic variation, POFAD, RAxML, Consensus Network, genetic divergence, *Tulipa*

## Abstract

Next Generation Sequencing (NGS) may enable estimating relationships among genotypes using allelic variation of multiple nuclear genes simultaneously. We explored the potential and caveats of this strategy in four genetically distant *Lilium* cultivars to estimate their genetic divergence from transcriptome sequences using three approaches: POFAD (Phylogeny of Organisms from Allelic Data, uses allelic information of sequence data), RAxML (Randomized Accelerated Maximum Likelihood, tree building based on concatenated consensus sequences) and Consensus Network (constructing a network summarizing among gene tree conflicts). Twenty six gene contigs were chosen based on the presence of orthologous sequences in all cultivars, seven of which also had an orthologous sequence in *Tulipa*, used as out-group. The three approaches generated the same topology. Although the resolution offered by these approaches is high, in this case there was no extra benefit in using allelic information. We conclude that these 26 genes can be widely applied to construct a species tree for the genus *Lilium*.

## Introduction

The preponderance of data used in plant molecular phylogenetics over the last decade comes from chloroplast DNA and nuclear rDNA (notably rDNA ITS) (APG, 2003, 2009; Chase and Reveal, 2009). Chloroplast DNA has the advantage of straightforward genetics: haploid, non-recombinant and highly conserved with respect to gene content and arrangement, notably among closely related species (Olmstead and Palmer, 1992). However, cpDNA reveals only half of the phylogenetic origin of a plant-lineage since it is uni-parentally inherited and its substitution rates are generally low compared with bi-parentally inherited nuclear DNA (Small et al., 2004). As a special case rDNA has been used extensively in Angiosperm (and fungal) phylogenetic reconstruction, especially using the Internal Transcribed Spacer regions (White et al., 1990; Baldwin, 1992). However, when not all rDNA copies are fully homogenized as was observed for instance in tulip and peonies (Sang et al., 1995; Booy et al., 2000; Lim et al., 2001; Alvarez and Wendel, 2003), the risk of using paralogs in phylogenetic reconstruction becomes large (Kim and Jansen, 1994; Sang et al., 1995; Alvarez and Wendel, 2003) and hence rDNA has been disregarded as phylogenetic marker in species-level Angiosperm phylogenetics [8]. Multi-locus, low copy nuclear DNA sequences have been used in plant phylogenetic studies since the late nineties (De La Torre et al., 2006; Hughes et al., 2006; Sanderson and McMahon, 2007; Griffin et al., 2011) and, because of their bi-parental inheritance and wealth of long and independently-inherited genes (Small et al., 2004), became the focus of plant phylogenetic reconstruction in general. Also, the ability to identify heterozygosity within individuals and hybrids (allelic variation) is considered a distinct advantage of using nuclear DNA over that from organelles. Using two alleles instead of one can give, in principle, better estimations of phylogenetic relationships between closely related taxa (Joly and Bruneau, 2006; Liu et al., 2008), or in case of species hybrids, enable establishing correct gene trees, in which both alleles are placed within the germplasm that they are derived from Zhang et al. (2013).

The availability of Next Generation Sequencing (NGS) data in plants opens the door to phylogenetic studies using a wide set of loci, representing truly genome-wide coverage. Commonly-used techniques for estimating phylogenetic trees from multiple-loci data are: concatenation or “super matrix” methods (Nylander et al., 2004), super tree construction (Beninda-Emonds, 2004) and gene tree parsimony (Page, 1998). On the other hand, *Bayesian Estimation of Species Trees* (Liu et al., 2008) and *Bayesian Evolutionary Analysis by Sampling Trees* (Drummond and Rambaut, 2007; Heled and Drummond, 2010) estimate species trees from separate gene trees and deal with the multi-allelic nature of genes by enabling incorporation of several genes separately in estimating effective population size and tree topology. This is implemented by using a Monte Carlo Markov Chain to find a posterior distribution of species trees. In this way concatenation is no longer necessary and differences in mutation rate between genes can be included in the analyses, or accommodated using appropriate priors. However, SNPs between the alleles are treated as ambiguous (IUPAC) bases in consensus sequences in this approach, obviously discarding part of the available data. Use of NGS data for phylogenetic reconstruction requires choices between trade-offs, in particular so when dealing with data derived from cultivated plants, which often have a complex genetic background that may or may not be well-documented.

Here we explore NGS data originally generated for genetic resource and SNP marker retrieval in *Lilium* (Shahin et al., 2012) in a phylogenetic context. *Lilium* L. was ranked among the top seven of the most popular flower bulb genera (Benschop et al., 2010). *Lilium* is classified into seven sections based on 13 morphological and two germination characteristics (Comber, 1949), and into four hybrid groups: Asiatic (A, *Sinomartagon* section), Oriental (O, *Archelirion* section), *Longiflorum* (L, *Leucolirion* subsection b), and Trumpet hybrid groups (T, *Leucolirion* subsection a). Phylogenetic relationships within *Lilium* were reconstructed using molecular markers (Dubouzet and Shinoda, 1999; Nishikawa et al., 1999, 2001; Arzate-Fernandez et al., 2005; Muratović et al., 2010). Most of the species clustered into clades correlating with their morphological classification of Comber (1949), but a few behaved differently. Species of section *Leucolirion* (subsection a and b) that were supposed to cluster closely according to Comber (Comber, 1949), grouped separately. Species of *Leucolirion* (subsection b) were closer to section *Sinomartagon*, and species of *Leucolirion* (subsection a) were closer to section *Archelirion* in both studies (Nishikawa et al., 1999; Arzate-Fernandez et al., 2005). Lily breeding dates back about 200 years (Shimizu, 1987), significant breakthroughs are only 50 years old however, starting with the breeding of Asiatic hybrids (McRae, 1998). It has only been since the 1970's that the lily has become, after tulip, the most important flower bulb and cut flower (Lim and Van Tuyl, 2006).

The aim of this study was to use transcriptome data for estimating both genetic divergence and relationships among four *Lilium* cultivars, and for comparing, for those orthologous sequences available, the data to a set of cultivars in *Tulipa*, the closest related cultivar group with transcriptome sequence data available (Shahin et al., 2012). We use three approaches that differ with respect to optimality criteria and type of data used and compare their results: (i) separate allelic data using distance analysis, as implemented in POFAD (Joly and Bruneau, 2006), (ii) concatenated analysis of consensed sequences, i.e., between the alleles, using maximum likelihood (RAxML, Stamatakis), and (iii) Consensus Networks (Holland et al., 2005) of separate parsimony gene trees derived from consensed sequences. Whereas RAxML is a tree building method, both the POFAD and Consensus Networks approach construct and visualize comparative data in networks. Consensus Networks are reconstructed by converting trees into splits to summarize possible among-tree conflict in a reticulate structure, where edge lengths are proportional to the occurrence of splits. In contrast, the POFAD algorithm calculates a pairwise distance matrix of all (separate) haplotypes, followed by conversion of this matrix into an organism-level distance matrix by taking the average of distances between the haploids. This matrix is then visualized in a Neighbor Network (Bryant and Moulton, 2004) allowing “non-treelike” patterns in the data. By using this algorithm we can combine the distance matrices of different loci without the need to concatenate the loci or to construct a (artificial) consensus allele per locus.

## Materials and methods

### Plant material

**Figure 1:**
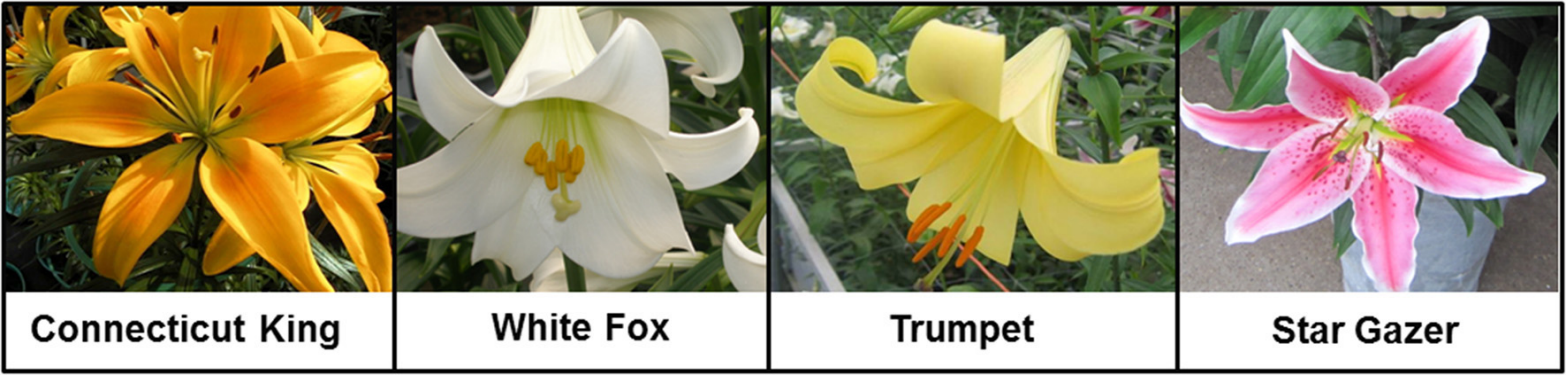
**Floral morphology of the four *Lilium* L. cultivars used in this study**.

Transcriptome sequence data of four *Lilium* and five *Tulipa* cultivars (all diploid) used for this study were from Shahin et al. (2012). The four *Lilium* cultivars, representing the four main hybrid groups of the genus *Lilium*, are: “Star Gazer” (Oriental, *Archelirion* section), breeding line “Trumpet 061099” (Trumpet, *Leucolirion* subsection a), “White Fox” (*Longiflorum, Leucolirion* subsection b), and “Connecticut King” (Asiatic, *Sinomartagon* section) (Figure 1). The five *Tulipa* cultivars are: “Cantata” and “Princeps,” which belong to *T. fosteriana* (*Eichleres* section), and “Bellona,” “Kees Nelis” and “Ile de France,” which belong to *T. gesneriana* (*Tulipa* section).

### Methodology

For RNA isolation, library processing and 454 sequencing protocols used see Shahin et al. (2012). The sequence data of the four *Lilium* cultivars were assembled using the CLC assembler (Shahin et al., 2012). As a result of the assembly step, an Ace file was generated that contains contigs (i.e., the consensus of all assembled ESTs that belong to one locus) which were used as starting point in this analysis. Contigs with high coverage (>100 reads per contig and at least 4 reads for each individual cultivar) were picked for further analysis. All the individual haplotype consensus sequences (e.g., Trumpet_A, Trumpet_B) for each gene were aligned in SeqMan and trimmed to the same size for all cultivars. If a contig showed more than two haplotypes/alleles per cultivar which indicates either assembled paralogs or sequencing errors, such contig was discarded. BlastX was used for annotation of contig consensus sequences. The number of polymorphic sites for each contig (27 contigs) were calculated using TOPALi v. 2 (Milne et al., 2009).

The 27 *Lilium* contigs were blasted to the Tulip-ALL assembly (BLASTn, 1E-20) (Shahin et al., 2012), in order to select tulip as out-group for subsequent tree building (see below). Seven of the 27 contigs showed to have orthologous sequence in the five *Tulipa* cultivars that have the same criteria (high coverage >100 reads per contig and at least 4 reads for each individual cultivar, and only 2 haplotypes per cultivar). These seven orthologous genes were analyzed using the same steps explained above. The number of polymorphic sites for each contig were calculated using TOPALi v. 2 (Milne et al., 2009).

### Recombination test

In order to use these gene contigs for phylogenetic or distance tree construction, recombination tests should be applied to avoid using any sequence that is putatively recombined, (e.g., Vriesendorp and Bakker, 2005). This was done on the different haplotypes within the cultivars using PDM (Probabilistic Divergence Measure) and DSS (Difference of Sum of Squares), both implemented in TOPALi v. 2 (Milne et al., 2009). The test operates by sliding a fixed-size window (e.g., 500 bp wide) along the alignment, comparing the left-hand part with the right-hand part in terms of phylogenetic topologies based on either part. In PDM the marginal posterior distributions of topologies are compared, whereas SSD fits pairwise genetic distances of each part to a phylogenetic tree based on the other part. Upon moving into a recombinant site, marginal distributions or SSD resp. should change. We used the default options of the program except for the nucleotide substitution model, where we replaced the (default) Jukes-Cantor model by Felsenstein84. Parametric bootstrapping was applied to estimate the significance of the predictions (100 reps). Observed values of DSS and PDM methods beyond the 95% point of this distribution may well correspond to a recombination event. Contigs with a putative recombination site were discarded for further study.

### Tree building and network analysis

#### POFAD

The edited and trimmed haplotypes of every locus were imported in MEGA 5 (Tamura et al., 2011) and an uncorrected genetic distance matrix (p-distance) was generated for each contig. Reweighting the individual matrices, which is essential to insure their equal contribution in the estimation of the genetic distance, was done by the algorithm implemented in POFAD (Joly and Bruneau, 2006). The genotypes' reweighted matrices for each gene contig individually was transferred to SplitsTree v.4 (Huson and Bryant, 2006) to construct Neighbor Networks. Similarly, the matrices of the 7 orthologous *Lilium* and *Tulipa* sequences were also transferred to SplitsTree v.4 for constructing Neighbor Networks.

#### RAxML

To compare the average distance-based POFAD output with that from a character-based tree-building analysis we first merged allelic/haplotype sequences for each individual cultivar by calculating their consensus (including IUPAC bases) using Bio Edit version 7 (http://www.mbio.ncsu.edu/BioEdit/bioedit.html), and then aligning them with other cultivars and concatenate the alignments of all contigs using Mesquite version 2.75 (Maddison and Maddison, 2011). The resulting super-matrix, see Supplementary Materials, was then analyzed in RAxML (Stamatakis et al., 2008) at the XSEDE Teragrid of the CIPRES science Gateway (Miller et al., 2010), including 100 replicates of fast-bootstrapping using the GTR-CAT substitution model (Stamatakis, 2006). Similarly, and to determine optimal rooting of our lily cultivars relationships, a super-matrix was generated for the seven genes orthologous between *Lilium* and *Tulipa* and then analyzed in RAxML (Stamatakis et al., 2008) using the same parameters.

#### Consensus network

The alignments of all gene contigs that were built using Mesquite version 2.75, were used to construct separate gene trees by standard heuristic search in PAUP^*^ (Swofford, 2003). All resulting parsimony trees, including multiple equally parsimonious trees from the same alignment, were pooled and input into SplitsTree v. 44 (Huson and Bryant, 2006), where they were decomposed into splits and assembled using the Consensus Network option. We applied various (split) conflict thresholds in order to assess among tree conflict.

## Results

From NGS sequences generated from leaf transcriptomes of the lily cultivars (Shahin et al., 2012), 27 contigs, with the highest overall sequence depth, were selected for this study. The length of these contigs ranged between 372 and 1230 bp (Table 1), and the number of polymorphic sites (on average) varied from one in contig_22926 to 71 in contig_36700 (Table 1). There were very few BlastX hits to known genes (Table 1, only the highest hit is shown). The total length of *Lilium* sequence data used for this study was 18,275 bp, containing 623 polymorphic sites, i.e., an average of one substitution event every 29 bp.

**Table 1:** **Description of the 27 *Lilium* contigs used in this study: length, informative sites (calculated using TOPALi), the top hit result of blasting them to gene bank is presented**.

**Contig ID**	**Length**	**Nr. polymorphic sites**	**Accession**	**Function: BLASTX**	***E*-value**
Contig_19510	372	26	CBI16691.3	unnamed protein product [*Vitis vinifera*]	7.00E-15
Contig_36292	378	6	XP_002489102.1	hypothetical protein SORBIDRAFT_1962s002010 [*Sorghum bicolor*]	9.00E-20
Contig_34203	408	15	XP_002271397.1	unknown [*Glycine max*]	1.00E-65
Contig_36290	423	6	XP_002488914.1	hypothetical protein SORBIDRAFT_0070s002020 [*Sorghum bicolor*]	3.00E-25
Contig_21012	490	43	CBI28652.3	unnamed protein product [*Vitis vinifera*]	1.00E-66
Contig_30305	500	12	XP_002322328.1	predicted protein [*Populus trichocarpa*]	1.00E-66
Contig_22926	510	1	CAN66875.1	hypothetical protein VITISV_009275 [*Vitis vinifera*]	6.00E-14
Contig_35696	551	32	CBI27136.3	unnamed protein product [*Vitis vinifera*]	3.00E-43
Contig_25751	588	11	ACI31551.1	heat shock protein 90-2 [*Glycine max*]	7.00E-91
Contig_48560	615	11	XP_002280853.1	hypothetical protein [*Vitis vinifera*]	8.00E-50
Contig_19882	630	40	NP_001060290.1	hypothetical protein OsJ_25146 [*Oryza sativa* Japonica Group]	7.00E-61
Contig_34918	634	41	XP_002460541.1	hypothetical protein SORBIDRAFT_02g030210 [*Sorghum bicolor*]	1.00E-33
Contig_36700	639	71	AAZ57445.1	lipoxygenase LOX2 [*Populus deltoides*]	5.00E-40
Contig_34983	660	18	NP_001183774.1	hypothetical protein LOC100502367 [*Zea mays*]	2.00E-113
Contig_34202	666	29	ACU18883.1	PREDICTED: hypothetical protein [*Vitis vinifera*]	6.00E-100
Contig_6165	714	3	YP_003587262.1	ATPase subunit 4 [*Citrullus lanatus*]	1.00E-71
Contig_21042	717	39	XP_002284696.1	PREDICTED: hypothetical protein [*Vitis vinifera*]	1.00E-62
Contig_5703	720	20	EEE57528.1	hypothetical protein OsJ_07840 [*Oryza sativa* Japonica Group]	6.00E-101
Contig_30546	729	49	CAN72815.1	hypothetical protein VITISV_004099 [*Vitis vinifera*]	6.00E-44
Contig_36051	736	12	EEC79215.1	hypothetical protein OsI_19939 [*Oryza sativa* Indica Group]	2.00E-125
Contig_72799	747	20	ACZ82298.1	cellulose synthase [*Phyllostachys edulis*]	2.00E-115
Contig_34429	817	24	AAY43222.1	cellulose synthase BoCesA5 [*Bambusa oldhamii*]	5.00E-121
Contig_20744	840	18	ABB46861.2	Enolase, putative, expressed [*Oryza sativa* Japonica Group]	2.00E-133
Contig_31438	957	10	ACG36494.1	histone mRNA exonuclease 1 [*Zea mays*]	2.00E-94
Contig_6081	987	2	AAV44205.1	unknow protein [*Oryza sativa* Japonica Group]	3.00E-62
Contig_6523	1017	29	AAW78691.1	peroxisomal acyl-CoA oxidase 1A [*Solanum cheesmaniae*]	3.00E-166
Contig_10364	1230	35	NP_001151315.1	transmembrane 9 superfamily protein member 1 [*Zea mays*]	0
Total	18,275	623				

Seven out of 27 contigs have orthologous sequences with five *Tulipa* cultivars, and were included in our study as a separate analysis (Table 2). The contig length ranged between 423 and 1230 bp. The number of polymorphic sites among the nine cultivars (on average) was very low in some contigs (8 sites in contig_6081), but much higher in others (200 sites in contig_10364) (Table 2). A total of 5790 bp with 587 polymorphic sites were available for this part of the study, of which 395 sites were polymorphic only between *Lilium* and *Tulipa*, 124 sites were also polymorphic within *Lilium*, and 68 were also polymorphic within *Tulipa*. This is equivalent to a substitution rate of 0.021 substitutions per site in *Lilium*, 0.012 in *Tulipa*, and 0.1 between *Lilium* and *Tulipa*.

**Table 2:** **As Table 1 but for the seven orthologous contigs between *Lilium* and *Tulipa* used in this study**.

***Lilium* Contig**	***Tulipa* Contig**	**Length**	**Nr. polymorphic sites**	**Function: BLASTX**	***E*-value**
Contig_36290	Contig_47963	423	17	hypothetical protein SORBIDRAFT_0070s002020 [*Sorghum bicolor*]	3.00E-25
Contig_34202	Contig_49866	666	86	PREDICTED: hypothetical protein [*Vitis vinifera*]	6.00E-100
Contig_5307	Contig_49304	720	87	hypothetical protein OsJ_07840 [*Oryza sativa* Japonica Group]	6.00E-101
Contig_72799	Contig_34429	747	69	cellulose synthase [*Phyllostachys edulis*]	2.00E-115
Contig_6081	Contig_29742	987	8	unknown protein [*Oryza sativa* Japonica Group]	3.00E-62
Contig_6523	Contig_48627	1017	120	peroxisomal acyl-CoA oxidase 1A [Solanum cheesmaniae]	3.00E-166
Contig_10364	Contig_55032	1230	200	transmembrane 9 superfamily protein member 1 [*Zea mays*]	0
Total		5790	587		

### Recombination test

In case a recombination event is detected in a contig this would indicate that more than one evolutionary history is present in this sequence. Therefore, any recombinant sequences, as detected by our TOPALi analysis, were discarded from further phylogenetic analysis. This turned out to be only one of the 27 *Lilium* contigs (contig_30546), which showed a possible recombination event between positions 157 and 220 bp.

### Tree building and network analysis

#### POFAD

**Figure 2:**
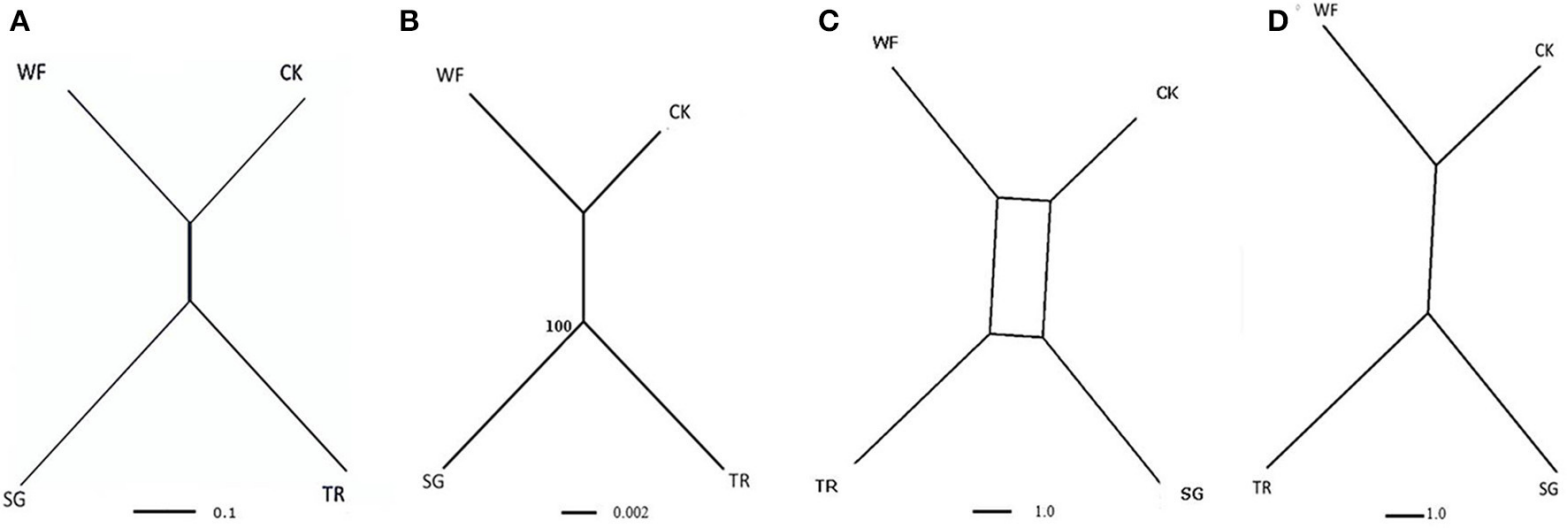
**Representation of the relationships of the four *Lilium* cultivars: “Star Gazer” referred to as “SG,” “Trumpet 061099” referred to as “TR,” “White Fox” referred to as “WF,” and “Connecticut King” referred to as “CK” obtained from the combined analysis of all 26 non-recombinant contigs. (A)** Neighbor Network based on 26 *Lilium* contigs, using the POFAD approach. **(B)** RAxML tree (with 100 rep. bootstrap support values) of the 26 *Lilium* concatenated consensus, **(C)** Consensus Network based on parsimony trees (see text) using a threshold of 0.33 split conflict, and **(D)** Consensus Network for the same trees using a threshold of 10%. Branch length is proportional to the genetic divergence among genotypes **(A,B**, with the scale bar indicating numbers of substitutions per site), and proportional to the occurrence of splits in the consensus network analysis **(D,C)**.

**Figure 3:**
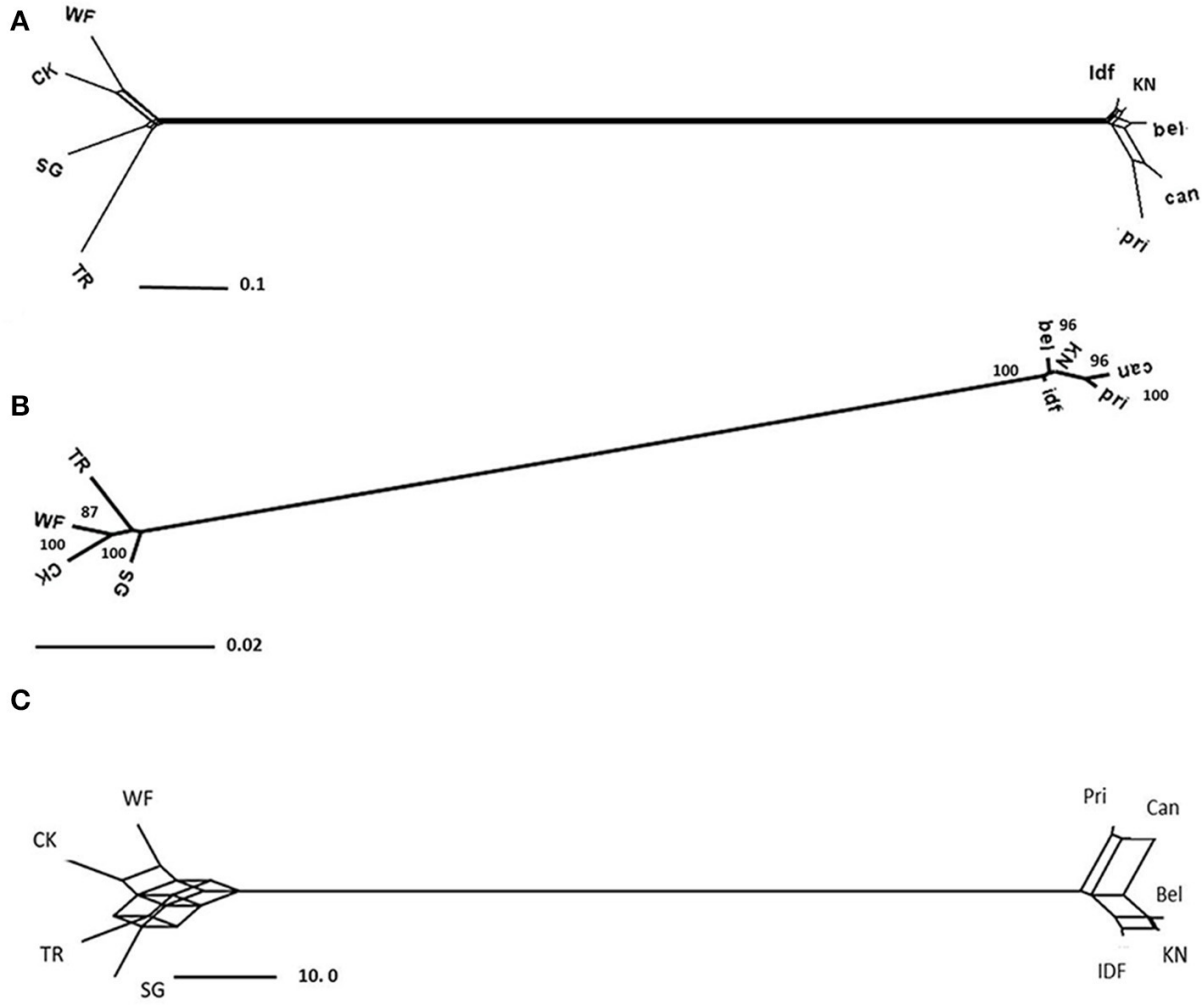
**As Figure 2 but with five *Tulipa* cultivars included (see text)**.

Gene trees were constructed for each contig separately using POFAD. In 23 of the 26 gene trees, “Connecticut King” and “White Fox” grouped together, as well as “Star Gazer” and “Trumpet” (the exceptions being Contig_25751, contig_6165, and contig_34202). The same clustering resulted from constructing the Neighbor Network of the combined weighted genetic distance matrices of the 26 gene contigs (Figure 2A). As expected, introducing *Tulipa* as an out-group to the analysis did not introduce changes in the clustering among the *Lilium* cultivars (Figure 3A). The four cultivars are connected to multiple edges in the Network (Figure 3A), possibly indicating “non tree-like” behavior of the sequences involved. As for *Tulipa*, “Cantata” and “Princeps” that belong to *T. fosteriana* grouped together and “Ile de France,” “Kees Nelis” and “Bellona” that belong to *T. gesneriana* clustered together as well with multiple edges (Figure 3A).

#### RAxML

RAxML analysis of the consensus sequences (alleles had been merged into consensus sequences, see M&M) of the concatenated 26 contigs (best tree) resulted in grouping “Connecticut King” and “White Fox” together without bootstrap support, and in grouping “Star Gazer” and “Trumpet” together with bootstrap value 100 (Figure 2B), yielding the same tree topology as POFAD Network. RAxML tree of the seven concatenated gene alignments of *Lilium* and *Tulipa* showed also a comparable topology and branch lengths as found using POFAD for both *Lilium* and *Tulipa* (Figure 3B) but with relatively high bootstrap values (Figure 3B).

#### Consensus network

After parsimony analyses of the separate 26 alignments (excluding the potentially recombinant contig_30546), all resulting 68 equally parsimonious reconstructions were combined in a Consensus Network that resulted in the same topology as the POFAD and RAxML tree (Figure 2C). Using a (default) split-conflict threshold of 0.33, the Consensus Network was tree-shaped (Figure 2D), whereas lowering this threshold to 5% resulted in a box structure separating the 4 cultivars at equidistance. For the seven ortholog analysis, for both all 4 *Lilium* and 5 *Tulipa* cultivars, we obtained a Consensus Network (Figure 3C) that was congruent with the POFAD Median Network showing comparable resolution.

## Discussion

Multi-locus genomic data obtained by NGS technology are rapidly becoming the main sources for inferring evolutionary relationships (Haussler et al., 2009; Emerson et al., 2010; Griffin et al., 2011) providing factors such as taxon sampling (Graybeal, 1998), polymorphic sites (Lopez et al., 2002) and hybridization (Naumov et al., 2000) have been properly accommodated. Nuclear DNA is more dynamic and evolves faster than plastid DNA, thus it provides a rich source of polymorphisms compared with plastid DNA; however, depending on what taxonomic level is targeted, we expect more nuclear genes to be required for constructing phylogenetic relationships due to the bi-parentally inherited and recombinant nature of nuclear DNA. In yeasts, a minimum of 8–20 genes were found to be sufficient to generate a stable species tree topology with bootstrap values of at least 70–95% (Rokas et al., 2003) which is largely influenced by number of informative sites present in these genes (Rokas et al., 2003) as well as the species tree estimation method used (Edwards et al., 2007). In our study, using 7 or 26 contigs genes resulted in the same tree topology. We did not further explore possible among gene tree incongruences as our Consensus Network analysis yielded patterns congruent or identical to those obtained by POFAD and RAxML. However, the use of TOPALi could in principle be extended from detecting candidate recombinant sites among haplotypes to the concatenated contigs, in order to detect among gene tree incongruence, when relevant. This could then provide valuable additional insight into the relationships between the cultivars.

Genetic variation among the four *Lilium* cultivars (0.034 substitutions per site) was higher than that detected for *Tulipa* (0.021 substitutions per site, data not shown). The same result was obtained using the seven orthologous genes between *Lilium* and *Tulipa*, which are expected to be conserved genes (0.021 subst/site in *Lilium* and 0.012 subst/site in *Tulipa*). As the cultivars used are thought to represent overall diversity and classification in these genera (see Introduction) we feel these rate differences are not affected by (taxonomic) sampling artifacts and may actually reflect genetic divergence within the respective genera.

Both *Lilium* and *Tulipa* are outcrossing species, and vegetatively reproduced. Thus, the apparent difference in evolutionary rate can probably be explained by generation time and breeding history. Generation time (2.5x faster in lily compared with tulip) is considered to be negatively correlated with substitution rate, while breeding and selection probably influences the fixation of substitutions over generations (Buschiazzo et al., 2012). In addition, sequence divergence rates are considered to be governed by life span, i.e., short-lived species are capable of changing more quickly than those that have a longer life span and reproduce less often, and indeed, higher evolution rates have been observed in annuals compared with perennials (Yue et al., 2010). Another possible explanation for the different evolutionary rates between *Tulipa* and *Lilium* is their breeding history, though documentation is limited due to the fact that breeding historically was widely done by amateurs and private companies before professional institutes took over (Benschop et al., 2010). However, it is known that the breeding history of lily is more complex than tulip since more species were involved, which might explain the difference in evolution rate between both cultivar groups, as it could reflect actual difference in *N*_e_.

In our analyses *Lilium* cultivars “Connecticut King” and “White Fox,” belonging to sections *Sinomartagon* and *Leucolirion* (subsection b) respectively, always grouped together, while “Star Gazer” and “Trumpet” (sections *Archelirion* and *Leucolirion* subsection a) clustered together as well (Figures 2A–D). Similar results were reported in other phylogenetic studies (Nishikawa et al., 1999; Arzate-Fernandez et al., 2005), based on cpDNA sequence comparisons. This is not in agreement with Comber's (Comber, 1949) classification, based on morphological and germination characteristics, in which “White Fox” and “Trumpet” belong to the same section *(Leucolirion)*. On the other hand, crosses of *Longiflorum* hybrids (L, *Leucolirion* subsection b) with Trumpet hybrids (T, *Leucolirion* subsection a) are less successful compared with crosses of Trumpet hybrids with Oriental hybrids (O, *Archelirion*) and compared to crosses of *Longiflorum* hybrids with Asiatic hybrids (*Sinomartagon*) (Alex van Silfhout, Wageningen UR Plant Breeding, personal observations). In the latter there are even combinations in which hybrids are fertile on the diploid level and can be used for analytic breeding (Khan et al., 2009). Thus, patterns derived from crossability and molecular markers appear to support each other in *Lilium*.

### Comparison of methods

Given the ongoing increase of generating comparative transcriptome data, at and below the plant species-level, comparing analytical approaches in terms of performance and accuracy is more important than ever. In this paper we demonstrate the relative performance of commonly-used tree and network building methods.

The POFAD algorithm implements allelic information for inferring genetic distances in cultivars. Using POFAD helped to include the variation between haplotypes in estimating their relationships by taking their average (i.e., un-observed) distances. However, the standard POFAD pipeline does not allow inferring node-support, for instance by bootstrap values. This could be overcome by bootstrapping the sequence alignments, then following the POFAD procedure for each bootstrapped (pseudo) alignment and summarizing the occurrence of groups (bootstrap frequencies), similar to Neighbor Joining bootstrapping.

Three lily gene contigs presented deviating Neighbor Joining topologies in our analyses. These reflect either artifacts due to the low number of samples used (long branches and short internode) (Wiens, 2005), the NJ algorithm itself, or biological deviation which can be explained by assuming that each genomic region underwent an unique array of evolutionary events such as recombination, selection, mutation and/or gene flow (Buerkle et al., 2011). If such fragments are highly informative for their own phylogenetic history, it might in principle be possible to track every genomic segment to its origin and thus visualize species hybridization events (Zhang et al., 2013).

The three approaches generated the same topology, be it at different resolutions. Neighbor Network and the Consensus Network approaches suggested some non-tree like evolution in our gene contig sequences, possibly reflecting reticulate breeding histories within *Lilium* and *Tulipa* (Figures 3A,C). On the other hand, the concatenated approach (RAxML) generated one tree that may actually simplify evolutionary history (Figure 3B).

Obviously, the limited “taxon” sampling of the cultivars used in our study could limit the generality of our findings. For instance, using bi-allelic data did not appear to add significantly to our estimation of cultivar relationships. It will be interesting to extend a comparative study using bi-allelic data of nDNA in order to assess evolutionary relationships between other, hybrid species, using these approaches. Limited “taxon” sampling combined with increased character-sampling can easily result in long-branch attraction artifacts (Wiens, 2005). However, our results in terms of topologies obtained by the three approaches was in agreement with Nishikawa et al. (1999), who used 55 *Lilium* species. This may be related to the availability of a large sequence data set rich in polymorphic sites (26 gene contigs sequences: more than 18 kb yielded around 600 polymorphic sites in *Lilium*) in the present study. These 26 contigs could therefore be an excellent set of genes to study the phylogeny of *Lilium* in depth by comparing to many other species and construct gene trees and species trees. Similarly, the seven orthologous sequences among the nine *Lilium* and *Tulipa* sequence provide promising material to build generic-level trees.

## Conclusions

Our study demonstrates the applicability of sequence data generated by next generation technology for estimating genetic divergence using the most commonly-used tree and network building methods. However, the benefit of the allelic nature of nuclear DNA in estimating the phylogeny of hybrids is still to be further established. The high number of polymorphic sites identified showed to be an effective tool for measuring genetic divergence, and the possible wide usage of these genes for phylogeny study for *Lilium* and *Tulipa* genus. The strategy to determine genetic distances based on a random set of genes for which orthologous sequences are retrieved from transcriptome sequencing, can be broadly applied. As the number of transcriptome datasets keeps increasing exponentially this will enable studies of the genetic relationships in many species complexes.

## Author contributions

Arwa Shahin participated in designing the work, generation, analysis and interpretation of the data. Freek T. Bakker contributed to the conception of the work, analysis and interpretation of the data. Jaap M. van Tuyl participated in the conception of the work. Marinus J. M Smulders participated in designing the work and interpretation of data. Paul Arens participated in the conception of the work and interpretation of data. The MS is written by Arwa Shahin, and revised critically by all co-authors. The co-authors approved the final version of the MS, and they agree to be accountable for all aspects of the work.

### Conflict of interest statement

The authors declare that the research was conducted in the absence of any commercial or financial relationships that could be construed as a potential conflict of interest.
